# Supplementary material for: Teacher-student collaborated multiple instance learning for pan-cancer PDL1 expression prediction from histopathology slides
Source: Nat Commun. 2024 Apr 9;15:3063. doi: 10.1038/s41467-024-46764-0 (PMC11004138; doi:10.1038/s41467-024-46764-0)
Supplement: Supplementary file 1 — Supplementary Information [file 41467_2024_46764_MOESM1_ESM.pdf]

Supplementary Note 1. Correlation analysis between model predictions and paired IHC slides.

To conduct the validation on IHC imaging, we collected 20 colon adenocarcinoma samples with paired H&E slides and IHC slides. In particular, we employed a color deconvolution technique [1] to separate the immunohistochemical staining from the hematoxylin counterstaining, allowing us to quantify PDL1 expression in the IHC slide images as shown in Fig. S1. The diaminobenzidine (DAB) channel was extracted from the original IHC tiles, and its pixel-wise intensity was utilized for the quantification of PDL1 expression. Subsequently, we performed image registration between the H&E and IHC slide pairs using SIFT [2] as the feature descriptor to mitigate any shifts and distortions (Fig. S2).

With the registered image pairs in place, we proceeded with regional-level correlation analysis. The entire slide images were partitioned into 100 regions, and Pearson's correlation coefficient was calculated by accumulating the predicted positive probability and diaminobenzidine intensity within each region. The predictions by the model trained at the tertile threshold with COAD FFPE data were adopted here.

Supplementary Note 2. Similarity measure between PDL1 relevant tumors and other tumors.

To explore the similarity between the aforementioned tumors and the PDL1-relevant ones, chi-square distribution was utilized to model the distribution of classification metrics values for PDL1-relevant tumors. A bootstrapping strategy with 100 random resamples was applied to each of the 9 tumors thus 900 subsets were constructed, whose classification metric values were utilized to construct the template matrix. Mahalanobis distance is used to measure the similarity between the cluster and each single point from the cluster. In this manner, the average distances between PDL1 relevant tumors and the template matrix at the threshold of median, upper tertile and upper quartile are respectively 1.849, 1.769 and 1.747. A chi-square distribution well fitted the aforementioned distance distribution, whose parameters could be found in supplementary Table S13. Corresponding P values through two-sided Kolmogorov-Smirnov (KS) test are 0.581, 0.172 and 0.190. The specific distributions are presented in supplementary Fig. S10. As the distance is regarded as continuous random variable, it is more appropriate to calculate corresponding probability density rather than the probability for individual data points. The probability density values listed in descending order are respectively 0.502 for HNSC, 0.481 for MESO, 0.450 for TGCT, 0.358 for UCS, 0.350 for THCA, 0.326 for READ, 0.280 for ACC, 0.280 for ESCA, 0.278 for OV, 0.258 for PRAD and 0.120 for LIHC as presented in Fig. S9, which could be taken as a similarity measure to the template matrix. Based on the measurement of correlation between morphological patterns and PDL1 mRNA expression levels, it was observed that some tumors such as HNSC, TGCT, THCA and READ still exhibit a high similarity to PDL1-relevant tumors. The observed differentiation in morpho-genomic links suggests that additional investigation is warranted for tumors with better morphological correlation. This implies that the intermediate processes connecting macroscopic changes in tumor morphology to microscopic gene expressions may share certain common features for these tumors and PDL1-relevant ones.

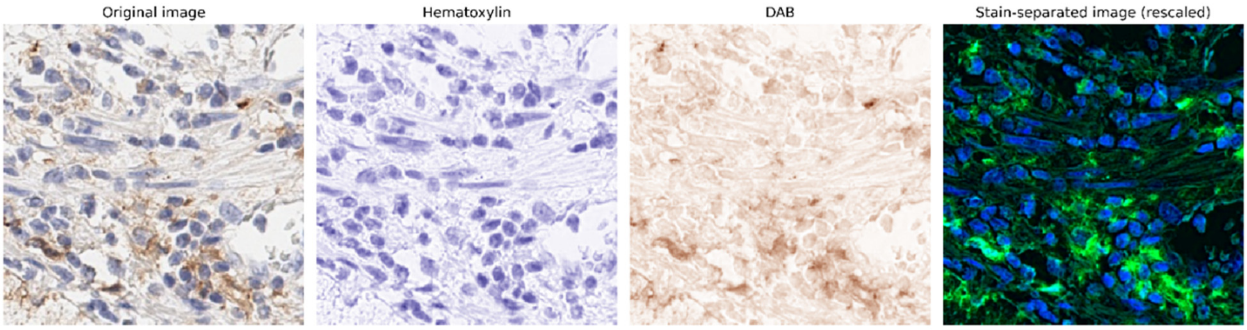

Fig. S1. The original IHC tile, hematoxylin channel, DAB channel and the pseudo fluorescence image generated by the two abovementioned channels.

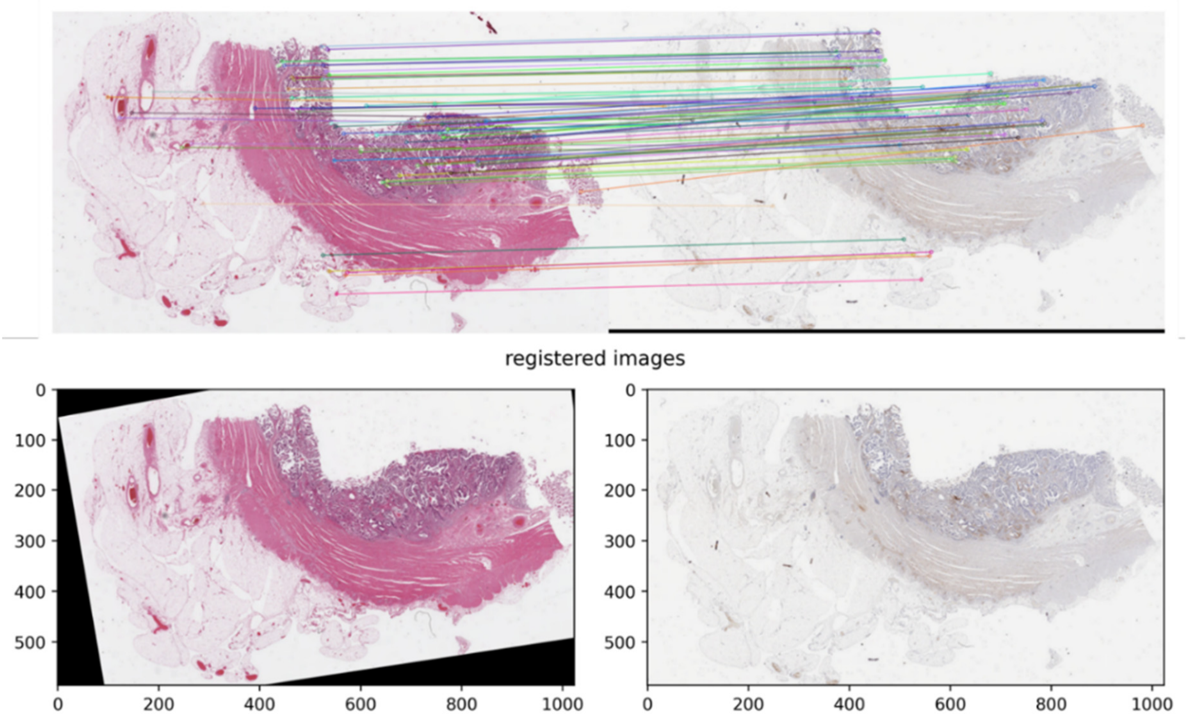

Fig. S2. Example of registration between the H&E slide image and the corresponding IHC slide image.

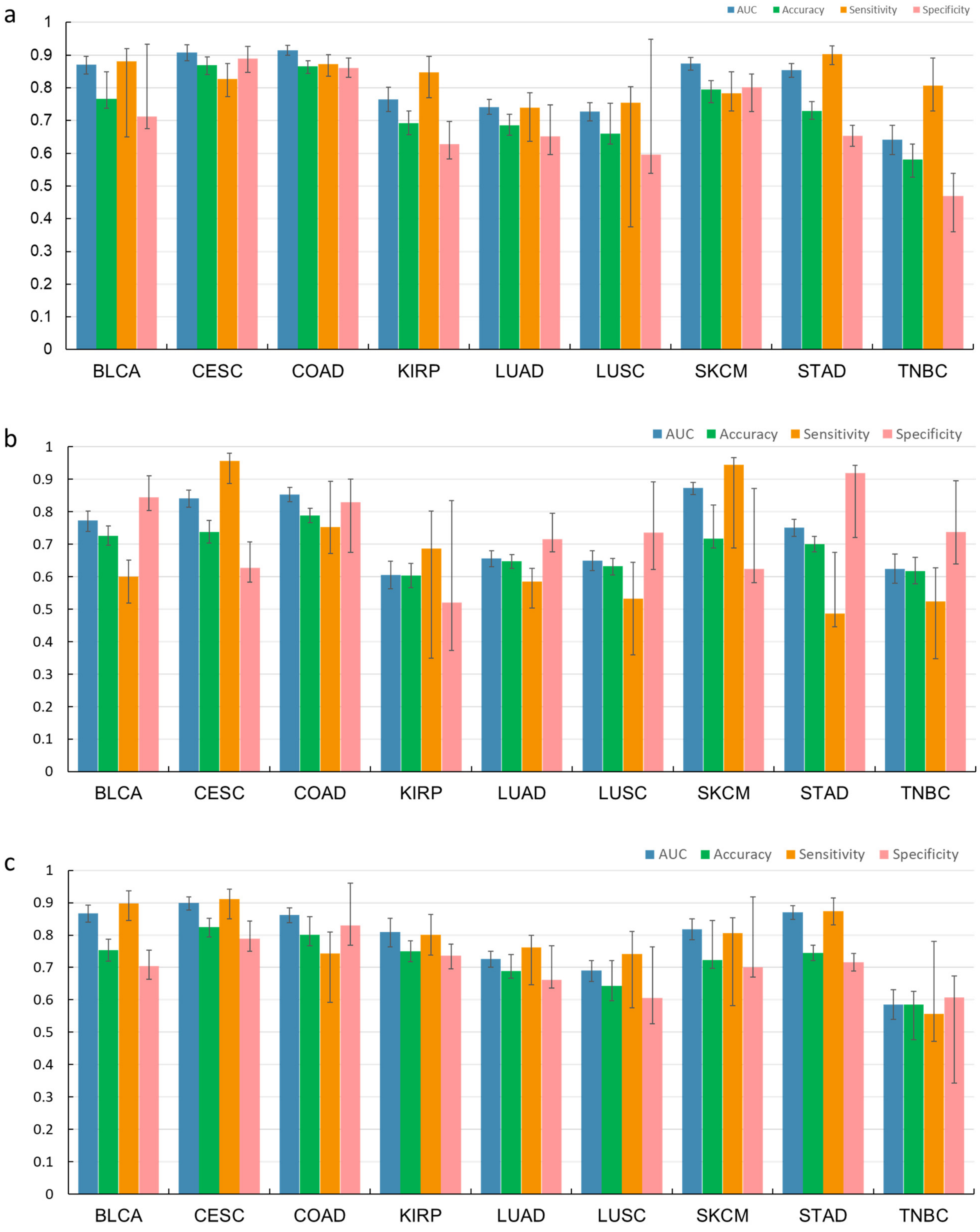

Fig. S3. Classification performance on PDL1 clinically relevant tumors obtained at different thresholds. **a**, Results at the threshold of upper tertile. **b**, Results at the threshold of median point. **c**, Results at the threshold of upper quartile. The data partitioning settings at the threshold of upper quarter and median point follow those at the threshold of upper tertile. Error bars represent 95% confidence intervals, calculated using bootstrap methods. Source data are provided as a Source Data file.

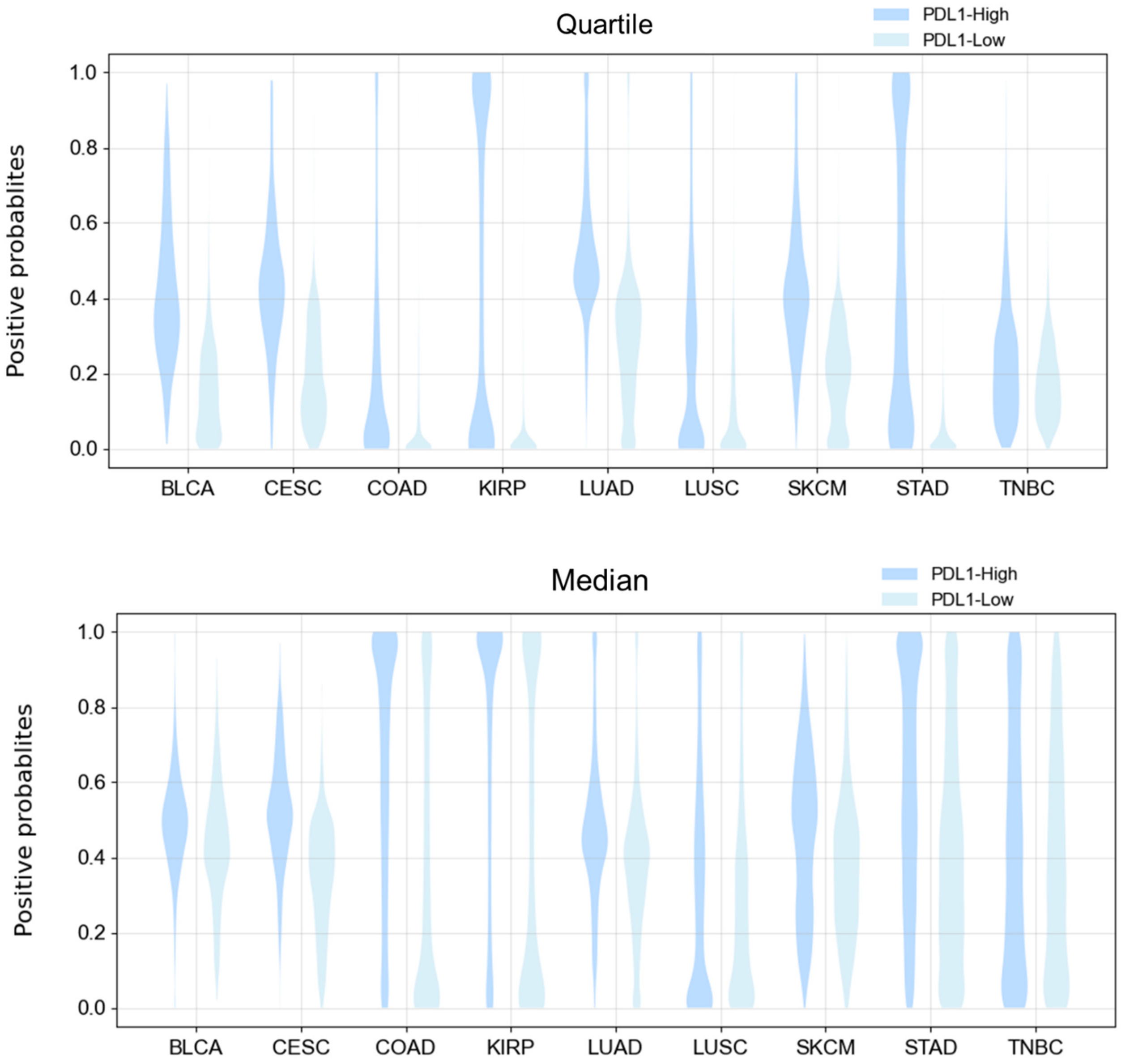

Fig. S4. Distribution of positive probability predicted by MILTS at the quartile and median thresholds on PDL1-relevant tumors. Source data are provided as a Source Data file.

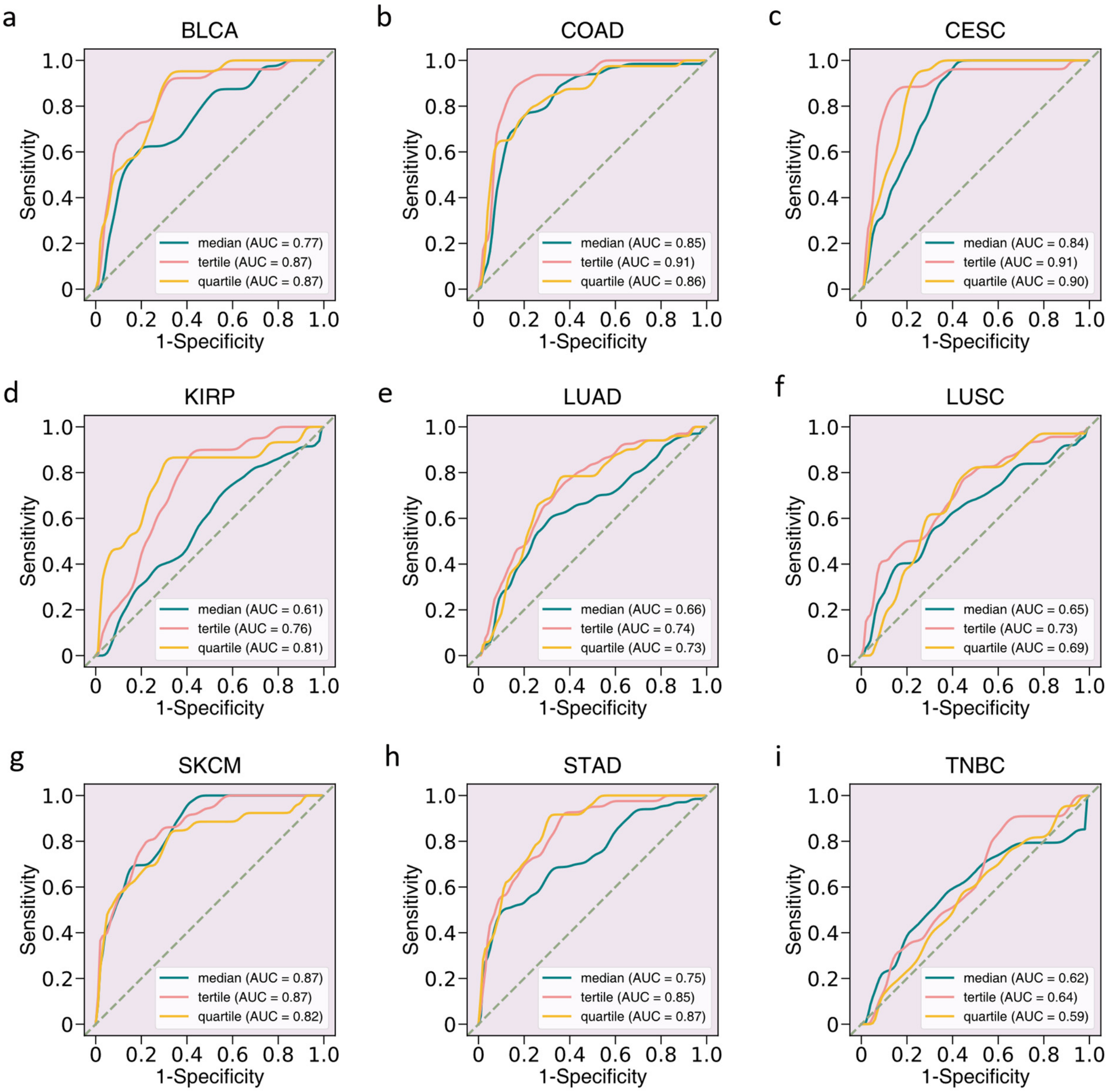

Fig. S5. Performance and results obtained by MILTS at different thresholds on clinically PDL1-relevant tumors. a-i, The corresponding ROC curves of tumors with PDL1 as an established biomarker with respect to different thresholds, where green, pink and yellow lines indicate respectively the performance achieved at the threshold of median, upper tertile and upper quartile. The results shown here only involve data of the test set.

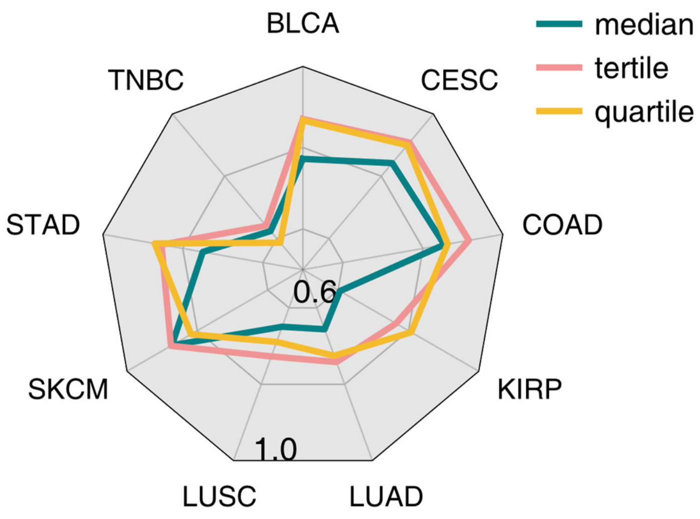

Fig. S6. AUC obtained by MILTS at different thresholds on clinically PDL1-relevant tumors. The radar chart demonstrating the AUC distribution in terms of different thresholds, where green, pink and yellow lines indicate respectively the performance achieved at the threshold of median, upper tertile and upper quartile. The results shown here only involve data of the test set. Source data are provided as a Source Data file.

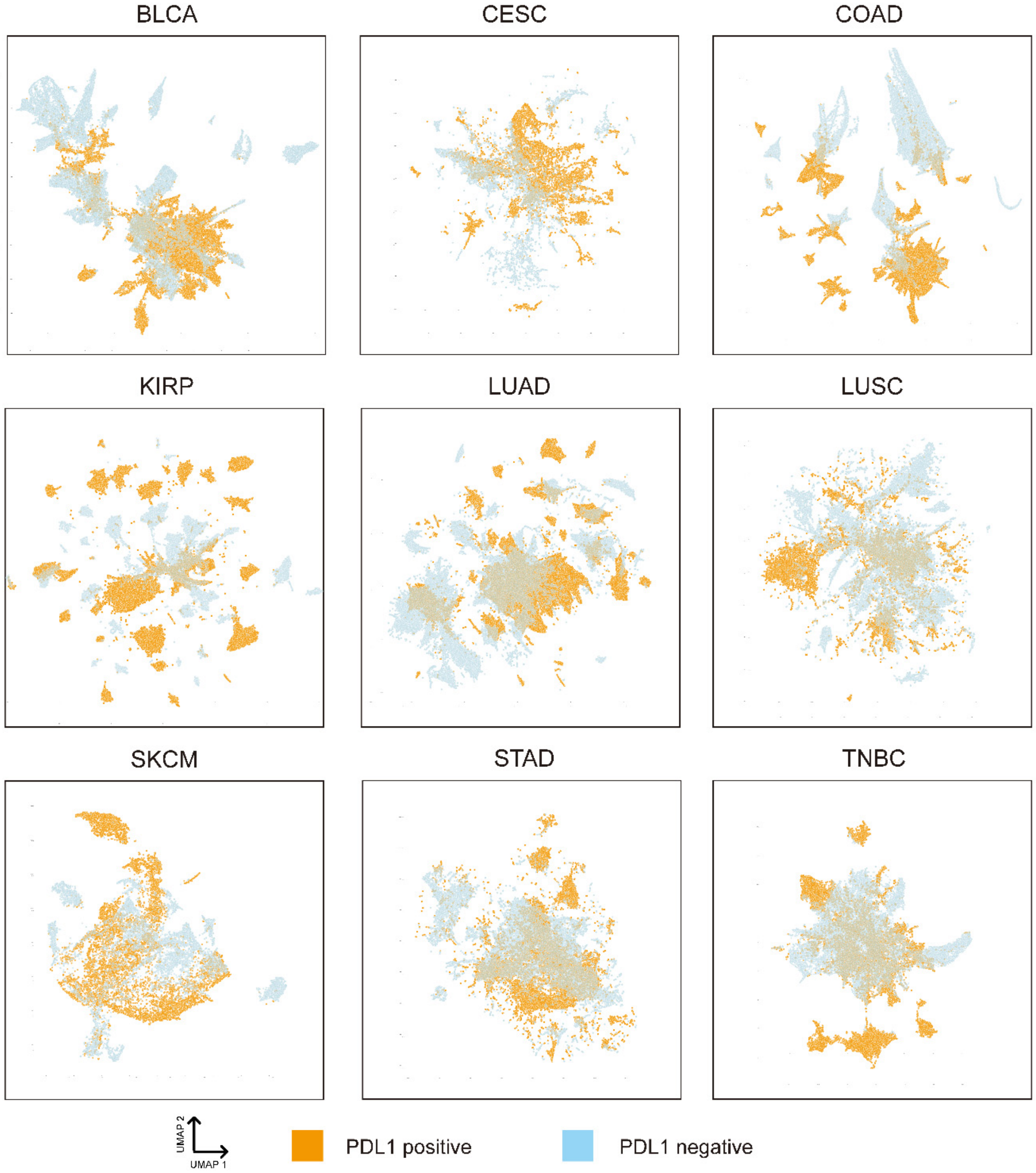

Fig. S7. Two-dimensional visualization by patch-level embeddings. Visualization of the representation space constructed by the patch-level classifier of MILTS for test set. The elements in orange indicate the instances from PDL1-high slides while the blue ones are from PDL1-low slides.

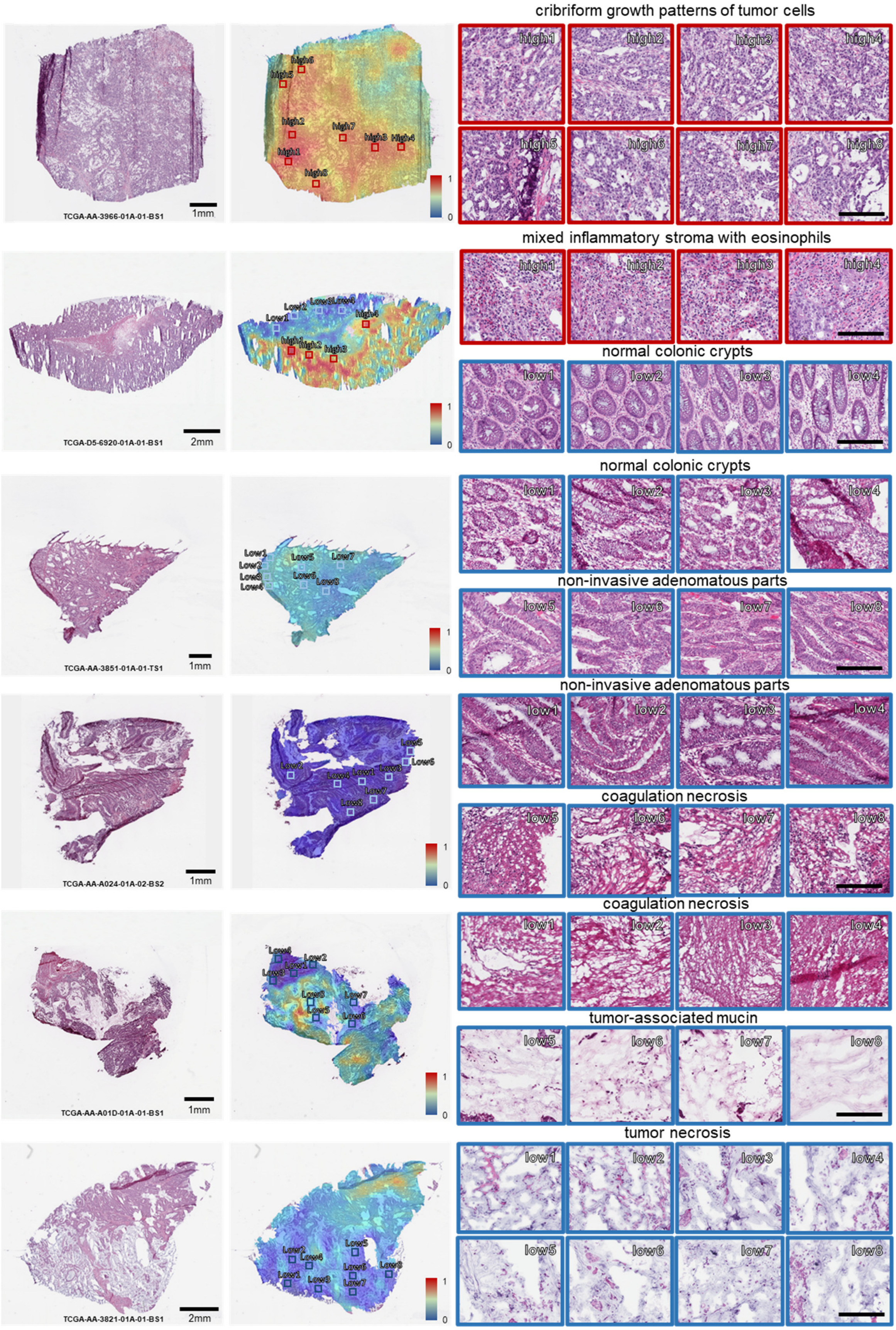

Fig. S8. Typical patterns for PDL1 high/low expression in fresh-frozen slides of COAD. Scale bars in the tile views: 100μm.

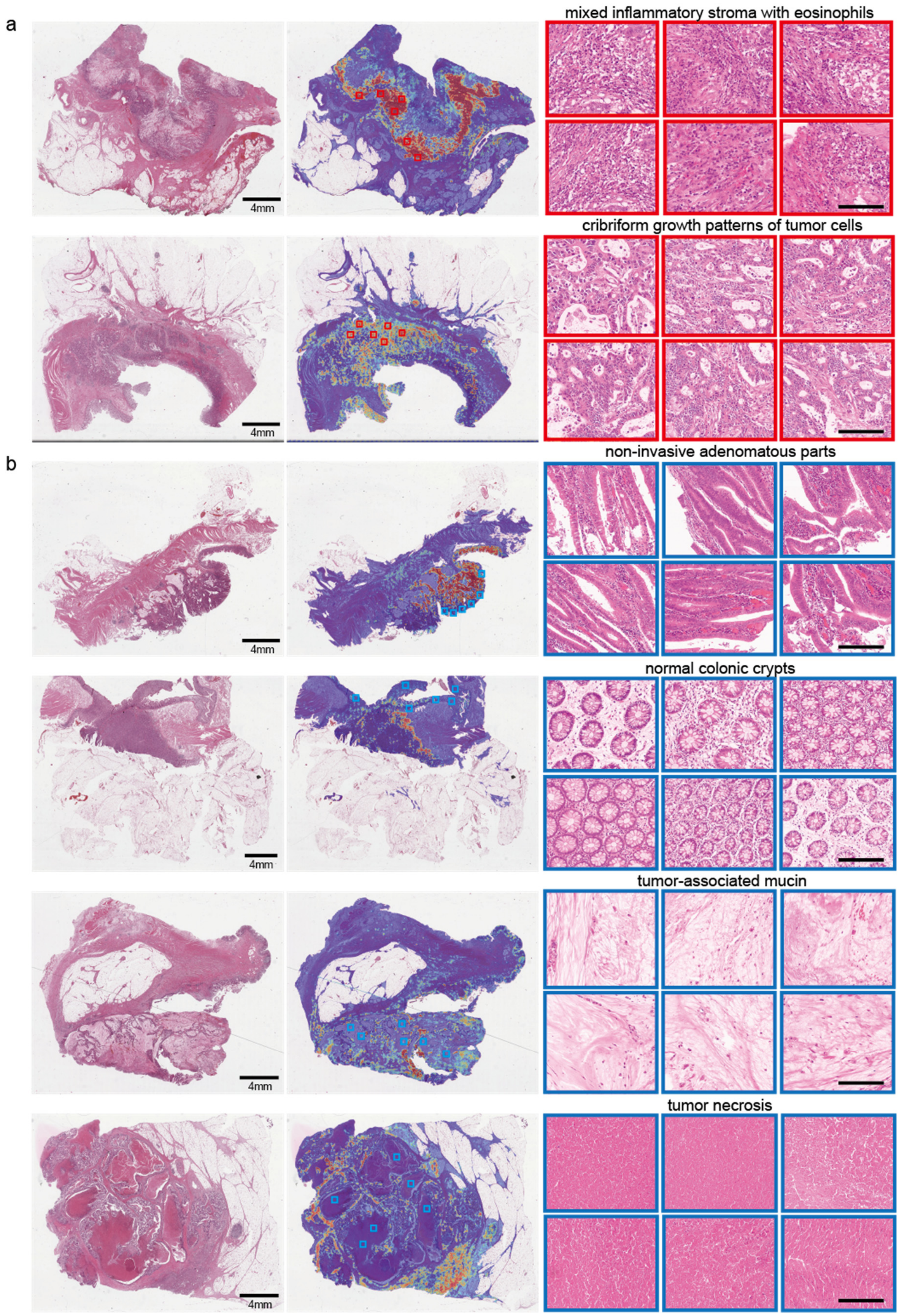

Fig. S9. Typical patterns for PDL1 high/low expression in FFPE slides of COAD. a, Example slides with typical PDL1 positive and **b,** negative patterns. Scale bars in the tile views of **a** and **b**: 100μm.

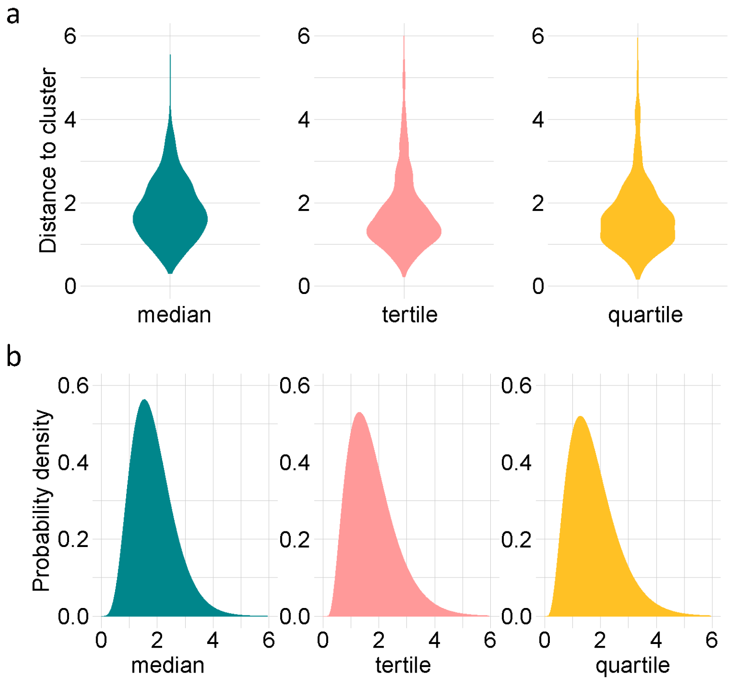

Fig. S10. The overall distribution of the distance between the PDL1 relevant tumors and the template cluster. a, The violin plot demonstrating the distribution of the Mahalanobis distance between the template cluster and PDL1-relevant ones at different thresholds. **b**, The probability density curves of the fitted chi-square distribution of the Mahalanobis distance between the template cluster and PDL1-relevant ones at different thresholds. Source data are provided as a Source Data file.

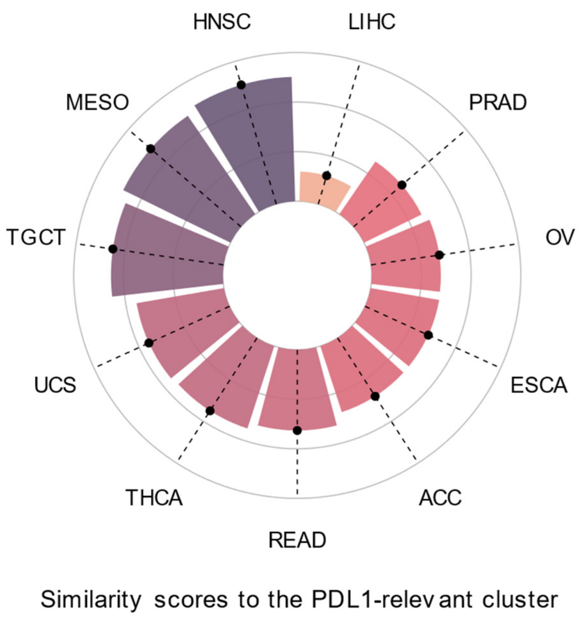

Fig. S11. Circular bar chart of probability density of tumors being PDL1 clinically-relevant using quantitative results obtained at the mean of three thresholds. Source data are provided as a Source Data file.

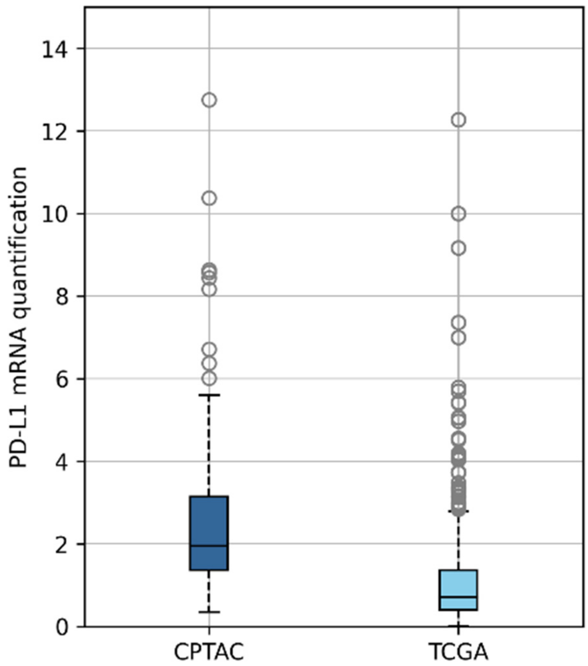

Fig. S12. mRNA distribution of CPTAC and TCGA COAD dataset. The central line in each box represents the median value. The box spans the interquartile range (IQR), with its lower and upper boundaries marking the 25th and 75th percentiles, respectively. Whiskers extend 1.5 times the interquartile range from the first and third quartiles. The sample sizes are respectively n=100 and n=451. Source data are provided as a Source Data file.

Table S1

QUANTILE VALUES FOR PDL1-RELEVANT TUMORS

Cancer type	BLCA	CESC	COAD	KIRP	LUAD	LUSC	SKCM	STAD	TNBC
Quartile	3.0	6.1	1.4	2.2	4.7	7.2	2.3	2.2	2.3
Tertile	2.0	4.2	1.1	1.6	3.8	5.0	1.7	1.8	2.0
Median	1.0	2.4	0.7	1.2	2.5	2.9	1.0	1.2	1.4

Table S2

QUANTILE VALUES FOR OTHER TUMORS

Cancer type	THCA	READ	HNSC	TGCT	MESO	PRAD	ESCA	UCEC	OV	ACC	LIHC
Quartile	3.6	1.1	5.0	2.6	2.4	0.8	2.6	0.8	1.1	0.5	0.6
Tertile	3.0	0.9	4.0	2.1	1.6	0.7	2.0	0.7	0.9	0.4	0.4
Median	2.2	0.6	2.4	1.3	1.0	0.6	1.4	0.6	0.6	0.3	0.3

Table S3

QUANTITATIVE RESULTS OF THE PROPOSED MODEL ON PDL1 CLINICALLY RELEVANT TUMORS AT MEDIAN THRESHOLD.

	LUAD	LUSC	BLCA	TNBC	CESC	STAD	COAD	KIRP	SKCM
AUC	0.656 (0.631~ 0.680)	0.650 (0.619~ 0.680)	0.773 (0.740~ 0.802)	0.624 (0.580~ 0.669)	0.841 (0.815~ 0.866)	0.751 (0.724~ 0.776)	0.854 (0.831~ 0.876)	0.605 (0.563~ 0.647)	0.872 (0.853~ 0.891)
Accuracy	0.648 (0.626~ 0.669)	0.633 (0.606~ 0.656)	0.726 (0.696~ 0.756)	0.617 (0.578~ 0.660)	0.737 (0.704~ 0.773)	0.701 (0.676~ 0.723)	0.789 (0.766~ 0.811)	0.603 (0.566~ 0.641)	0.718 (0.689~ 0.821)
Sensitivity	0.585 (0.503~ 0.625)	0.532 (0.359~ 0.644)	0.599 (0.518~ 0.650)	0.523 (0.348~ 0.627)	0.957 (0.886~ 0.980)	0.487 (0.445~ 0.676)	0.753 (0.671~ 0.894)	0.687 (0.348~ 0.801)	0.945 (0.688~ 0.967)
Specificity	0.716 (0.676~ 0.795)	0.736 (0.622~ 0.893)	0.844 (0.803~ 0.910)	0.738 (0.639~ 0.895)	0.628 (0.583~ 0.707)	0.918 (0.720~ 0.943)	0.829 (0.674~ 0.900)	0.521 (0.373~ 0.835)	0.623 (0.582~ 0.872)

Data enclosed in the parentheses represent the corresponding lower bound and the upper bound of 95% confidence interval. The intervals were obtained by bootstrapping strategy with 2000 random resamples.

Table S4

QUANTITATIVE RESULTS OF THE PROPOSED MODEL ON PDL1 CLINICALLY RELEVANT TUMORS AT THE THRESHOLD OF UPPER QUARTILE.

	LUAD	LUSC	BLCA	TNBC	CESC	STAD	COAD	KIRP	SKCM
AUC	0.725 (0.700~ 0.750)	0.690 (0.657~ 0.720)	0.868 (0.841~ 0.892)	0.586 (0.540~ 0.631)	0.899 (0.878~ 0.918)	0.870 (0.848~ 0.890)	0.862 (0.839~ 0.883)	0.809 (0.764~ 0.851)	0.817 (0.785~ 0.850)
Accuracy	0.689 (0.668~ 0.739)	0.644 (0.597~ 0.722)	0.754 (0.720~ 0.787)	0.586 (0.476~ 0.625)	0.824 (0.794~ 0.852)	0.744 (0.721~ 0.769)	0.802 (0.767~ 0.85)	0.750 (0.718~ 0.782)	0.723 (0.697~ 0.845)
Sensitivity	0.762 (0.647~ 0.800)	0.741 (0.575~ 0.811)	0.898 (0.845~ 0.936)	0.557 (0.472~ 0.781)	0.911 (0.850~ 0.942)	0.874 (0.831~ 0.914)	0.743 (0.592~ 0.809)	0.801 (0.739~ 0.864)	0.806 (0.582~ 0.854)
Specificity	0.662 (0.636~ 0.767)	0.606 (0.525~ 0.763)	0.704 (0.663~ 0.754)	0.607 (0.343~ 0.674)	0.788 (0.750~ 0.843)	0.716 (0.689~ 0.743)	0.830 (0.769~ 0.960)	0.736 (0.697~ 0.772)	0.700 (0.669~, 0.919)

Data enclosed in the parentheses represent the corresponding lower bound and the upper bound of 95% confidence interval. The intervals were obtained by bootstrapping strategy with 2000 random resamples.

Table S5

QUANTITATIVE RESULTS OF THE PROPOSED MODEL ON FFPE SLIDES AT TERTILE THRESHOLD.

	BLCA	CESC	COAD	KIRP	LUAD	LUSC	SKCM	STAD	TNBC
AUC	0.81	0.80	0.84	0.83	0.71	0.61	0.71	0.82	0.54
Accuracy	0.76	0.75	0.82	0.81	0.67	0.61	0.62	0.79	0.61
Sensitivity	0.78	0.77	0.67	0.67	0.72	0.54	0.82	0.70	0.54
Specificity	0.77	0.73	0.94	0.84	0.63	0.70	0.52	0.80	0.62

Table S6

QUANTITATIVE RESULTS ON FFPE SLIDES USING THE MODEL TRAINED WITH FRESH-FROZEN SLIDES.

	BLCA	CESC	COAD	KIRP	LUAD	LUSC	SKCM	STAD	TNBC
AUC	0.68	0.73	0.72	0.31	0.64	0.67	0.68	0.80	0.50
Accuracy	0.63	0.64	0.67	0.59	0.65	0.68	0.65	0.74	0.61
Sensitivity	0.79	0.89	0.80	0.31	0.70	0.63	0.73	0.76	0.38
Specificity	0.52	0.49	0.57	0.71	0.62	0.71	0.64	0.73	0.74

Table S7

HYPERPARAMETER SETTINGS OF COMPARISON METHODS

Methods	Optimizer	Learning rate	Batch size	Patch size
Campella's	ADAM<br>β_1=0.9, β_2=0.999, ϵ=1e-8	1e-4	512	256×256
TransMIL	Rectified Adam<br>β_1=0.9, β_2=0.999, ϵ=1e-8	2e-4	1	256×256
CLAM	ADAM<br>β_1=0.9, β_2=0.999, ϵ=1e-8	1e-4	1	256×256

Table S8

Quantitative Results of The Proposed Model on Other Tumors at The Threshold of Upper Quartile.

	ACC	ESCA	HNSC	LIHC	MESO	OV	PRAD	READ	TCGT	THCA	UCEC
AUC	0.819	0.496	0.719	0.558	0.707	0.644	0.629	0.828	0.763	0.838	0.641
Accuracy	0.707	0.560	0.630	0.637	0.677	0.584	0.501	0.680	0.811	0.779	0.526
Sensitivity	0.800	0.650	0.790	0.459	0.625	0.664	0.786	0.832	0.693	0.769	0.777
Specificity	0.685	0.514	0.584	0.692	0.697	0.544	0.417	0.651	0.850	0.783	0.439

Table S9

QUANTITATIVE RESULTS OF THE PROPOSED MODEL ON OTHER TUMORS AT THE THRESHOLD OF UPPER TERTILE.

	ACC	ESCA	HNSC	LIHC	MESO	OV	PRAD	READ	TCGT	THCA	UCEC
AUC	0.550	0.621	0.736	0.463	0.673	0.596	0.657	0.818	0.719	0.818	0.614
Accuracy	0.548	0.596	0.682	0.417	0.619	0.524	0.689	0.765	0.736	0.728	0.590
Sensitivity	0.733	0.787	0.830	0.934	0.738	0.881	0.471	0.750	0.632	0.831	0.662
Specificity	0.453	0.488	0.623	0.132	0.542	0.286	0.799	0.769	0.794	0.676	0.555

Table S10

QUANTITATIVE RESULTS OF THE PROPOSED MODEL ON OTHER TUMORS AT THE THRESHOLD OF MEDIAN.

	ACC	ESCA	HNSC	LIHC	MESO	OV	PRAD	READ	TCGT	THCA	UCEC
AUC	0.535	0.646	0.691	0.493	0.672	0.596	0.669	0.804	0.796	0.833	0.638
Accuracy	0.556	0.640	0.658	0.559	0.619	0.612	0.629	0.760	0.736	0.787	0.619
Sensitivity	0.541	0.374	0.545	0.583	0.736	0.696	0.816	0.808	0.563	0.813	0.739
Specificity	0.543	0.937	0.723	0.523	0.542	0.520	0.454	0.741	0.961	0.765	0.505

Table S11

PATIENT CHARACTERISTICS OF CPTAC COHORT

Dataset	CPATAC-COAD (n=100)	CPTAC-BRCA (n=117)
Age (year)		
Range	/	31.3-91.3
Average	/	60.7
Sex (n, %)		
Female	58 (58)	106 (90.6)
Male	42 (42)	/
Not reported	/	11 (9.4)
Tumor Stage (n, %)		
Stage I	10 (10)	4 (3.4)
Stage II	41 (41)	70 (59.8)
Stage III	42 (42)	32 (27.4)
Stage IV	7 (7)	/
Not performed/reported	/	11 (9.4)

Table S12

PARAMETER SETTINGS FOR DATA AUGMENTATION

Methods	RandomVerticalFlip	RandomRotation	RandomResizedCrop	ColorJitter	Normalize
parameters	DEFAULT	Degree = (-90°, 90)	Size = (224, 224)	brightness=0.2, contrast=0.2, saturation=0.2, hue=0.2	mean=(0.485, 0.456, 0.406) std=(0.229, 0.224, 0.225)

Table S13

FITTED PARAMETERS OF CHI-SQUARE DISTRIBUTION

Threshold	Degrees of freedom	Scale factor	Shifting
Upper quartile	11.898	0.156	-0.011
Upper tertile	6.794	0.234	0.178
Median	6.981	0.235	0.108

SUPPLEMENTARY REFERENCES

[1] Ruifrok, A. C., & Johnston, D. A. Quantification of histochemical staining by color deconvolution. *Analytical and quantitative cytology and histology* **23**, 291-299 (2001).

[2] Lowe, D. G. Distinctive image features from scale-invariant keypoints. *International journal of computer vision* **60**, 91-110 (2004).
