# Supplementary material for: Teacher-student collaborated multiple instance learning for pan-cancer PDL1 expression prediction from histopathology slides
Source: Nat Commun. 2024 Apr 9;15:3063. doi: 10.1038/s41467-024-46764-0 (PMC11004138; doi:10.1038/s41467-024-46764-0)
Supplement: Supplementary file 3 — Reporting Summary [file 41467_2024_46764_MOESM3_ESM.pdf]

nature portfolio

Corresponding author(s): Thomas Grünewald, Moritz Gerstung, Xiangzhi Bai

Last updated by author(s): Jan 30, 2024

Reporting Summary

Nature Portfolio wishes to improve the reproducibility of the work that we publish. This form provides structure for consistency and transparency in reporting. For further information on Nature Portfolio policies, see our Editorial Policies and the Editorial Policy Checklist.

Statistics

For all statistical analyses, confirm that the following items are present in the figure legend, table legend, main text, or Methods section.

n/a	Confirmed	
☐	☒	The exact sample size (n) for each experimental group/condition, given as a discrete number and unit of measurement
☐	☒	A statement on whether measurements were taken from distinct samples or whether the same sample was measured repeatedly
☐	☒	The statistical test(s) used AND whether they are one- or two-sided<br>*Only common tests should be described solely by name; describe more complex techniques in the Methods section.*
☒	☐	A description of all covariates tested
☒	☐	A description of any assumptions or corrections, such as tests of normality and adjustment for multiple comparisons
☐	☒	A full description of the statistical parameters including central tendency (e.g. means) or other basic estimates (e.g. regression coefficient) AND variation (e.g. standard deviation) or associated estimates of uncertainty (e.g. confidence intervals)
☐	☒	For null hypothesis testing, the test statistic (e.g. F, t, r) with confidence intervals, effect sizes, degrees of freedom and P value noted<br>*Give P values as exact values whenever suitable.*
☒	☐	For Bayesian analysis, information on the choice of priors and Markov chain Monte Carlo settings
☒	☐	For hierarchical and complex designs, identification of the appropriate level for tests and full reporting of outcomes
☒	☐	Estimates of effect sizes (e.g. Cohen's d, Pearson's r), indicating how they were calculated

Our web collection on statistics for biologists contains articles on many of the points above.

Software and code

Policy information about availability of computer code

Data collection	Data for this study was collected from TCGA and CPTAC, which are publicly available. The TCGA data can be accessed and downloaded from the TCGA Data Portal using GDC Data Transfer Tool (v1.6.1). The CPTAC image data can be downloaded from The Cancer Imaging Archive using IBM Aspera Connect (3.11.2.63).
Data analysis	The code of this study are available through a Code Ocean compute capsule in https://codeocean.com/capsule/9197393/tree/v1. Algorithms were mostly programmed with Python (version 3.8.10) and libraries mainly involved were h5py==3.5.0, numpy==1.19.5, nystrom-attention==0.0.11, opencv-python==4.5.3, openslide-python==1.1.2, pillow==8.4.0, pytorch-lightning==1.5.10, scikit-image==0.18.3, scikit-learn==1.13.0, timm==0.4.12, torch==1.9.1, torchvision==0.10.1 and yacs=0.1.8.

For manuscripts utilizing custom algorithms or software that are central to the research but not yet described in published literature, software must be made available to editors and reviewers. We strongly encourage code deposition in a community repository (e.g. GitHub). See the Nature Portfolio guidelines for submitting code & software for further information.

Data

Policy information about availability of data

All manuscripts must include a data availability statement. This statement should provide the following information, where applicable:

- Accession codes, unique identifiers, or web links for publicly available datasets
- A description of any restrictions on data availability
- For clinical datasets or third party data, please ensure that the statement adheres to our policy

TCGA data (images, as well as transcriptomic and clinical data) are publicly available from http://gdc.cancer.gov. CPTAC image data are publicly available from https://wiki.cancerimagingarchive.net/display/Public/CPTAC+Imaging+Proteomics and transcriptomic data from http://gdc.cancer.gov. The paired H&E slide and IHC slide images from Charité–Universitätsmedizin Berlin in this study are accessible upon request.

Research involving human participants, their data, or biological material

Policy information about studies with human participants or human data. See also policy information about sex, gender (identity/presentation), and sexual orientation and race, ethnicity and racism.

Reporting on sex and gender	Data for this study was collected from TCGA and CPTAC, which include this information as part of their publicly available clinical data. We did not independently collect, modify, or interpret these variables. Age and sex were not considered for this analysis and may not be published due to regulatory reasons. Colon cancer demonstrates no substantial sex-specific characteristics, and PDL1, a widely recognized biomarker, is established as independent of both sex and age factors. Therefore, these metadata were not utilized in producing the research results of this paper, and have no effect on the interpretation and the reproducibility of results.
Reporting on race, ethnicity, or other socially relevant groupings	Data for this study was collected from TCGA and CPTAC, which provide this information as part of their publicly available clinical data. We did not independently collect, modify, or interpret these demographic variables. Information on race, ethnicity and other socially relevant groupings was not collected for the Charité cohort.
Population characteristics	Data for this study was collected from TCGA and CPTAC, which provide this data within its publicly accessible clinical data. Our study did not involve the independent collection, alteration, or interpretation of these population characteristics. In the Charité cohort, there were 15 individuals with microsatellite stability (MSS) and 5 with microsatellite instability (MSI).
Recruitment	N/A
Ethics oversight	Analyses on the Charité cohort were done on irreversibly anonymized samples without relevant metadata or genetic information, which precludes re-identification. This study was approved by the Ethics Committee of the Charité University Medicine (#EA4/046/21) and complied with all relevant ethical regulations. Informed consent from all participants were obtained. Human participants did not receive financial or any other compensation.

Note that full information on the approval of the study protocol must also be provided in the manuscript.

Field-specific reporting

Please select the one below that is the best fit for your research. If you are not sure, read the appropriate sections before making your selection.

☒ Life sciences ☐ Behavioural & social sciences ☐ Ecological, evolutionary & environmental sciences

For a reference copy of the document with all sections, see nature.com/documents/nr-reporting-summary-flat.pdf

Life sciences study design

All studies must disclose on these points even when the disclosure is negative.

Sample size	Sample sizes were derived from the maximum number of cases available in specific cancer types in the TCGA dataset. 20 pairs of samples were collected from Charité–Universitätsmedizin Berlin for IHC validation. The sample size choice of 20 pairs of HE and IHC slides was empirically guided, factoring in the limited availability of these paired specimens and aligning with normative sample sizes for initial studies in our field.
Data exclusions	N/A
Replication	The codes for replication including training and testing of MILTS are available through a Code Ocean compute capsule in https://codeocean.com/capsule/9197393/tree/v1.
Randomization	Samples were randomly assigned to the training, validation and test sets.
Blinding	Investigators were blinded to group allocation during data collection and analysis.

Reporting for specific materials, systems and methods

We require information from authors about some types of materials, experimental systems and methods used in many studies. Here, indicate whether each material, system or method listed is relevant to your study. If you are not sure if a list item applies to your research, read the appropriate section before selecting a response.

Materials & experimental systems

n/a	Involved in the study
☒	☐ Antibodies
☒	☐ Eukaryotic cell lines
☒	☐ Palaeontology and archaeology
☒	☐ Animals and other organisms
☒	☐ Clinical data
☒	☐ Dual use research of concern
☒	☐ Plants

Methods

n/a	Involved in the study
☒	☐ ChIP-seq
☒	☐ Flow cytometry
☒	☐ MRI-based neuroimaging
